# Patterns and correlates of major depression in Chinese adults: a cross-sectional study of 0.5 million men and women

**DOI:** 10.1017/S0033291716002889

**Published:** 2016-12-06

**Authors:** Y. Chen, D. Bennett, R. Clarke, Y. Guo, C. Yu, Z. Bian, L. Ma, Y. Huang, Q. Sun, N. Zhang, X. Zheng, J. Chen, R. Peto, K. S. Kendler, L. Li, Z. Chen

**Affiliations:** 1 Nuffield Department of Population Health, Medical Research Council Population Health Research Unit (MRC PHRU) & Clinical Trial Service Unit and Epidemiological Studies Unit (CTSU), University of Oxford, Oxford, UK; 2 Nuffield Department of Population Health, Clinical Trial Service Unit & Epidemiological Studies Unit (CTSU), University of Oxford, Oxford, UK; 3 Chinese Academy of Medical Sciences, Dong Cheng District, Beijing, People's Republic of China; 4 Department of Epidemiology and Biostatistics, School of Public Health, Peking University Health Science Center, Beijing, People's Republic of China; 5 Suzhou CDC, 72 Sanxiang Road, Suzhou, People's Republic of China; 6 Guanxi Provincial CDC, 18 Jinzhou Road, Nanning, Guanxi, People's Republic of China; 7 Pengzhou CDC, 331 Longta Road, Pengzhou, Sichuan, People's Republic of China; 8 Sichuan Provincial CDC, 6 Chendu City Middle School Road, Chendu, People's Republic of China; 9 Meilan CDC, 70 Meilan District, Haikou, People's Republic of China; 10 China National Center for Food Safety Risk Assessment, Chaoyang District, Beijing, People's Republic of China; 11 Department of Psychiatry, Virginia Commonwealth University, Virginia Institute for Psychiatric and Behavioural Genetics, Richmond, VA, USA

**Keywords:** China rural regions, family conflict, living alone, major depressive disorder, stressful life events

## Abstract

**Background:**

Worldwide 350 million people suffer from major depression, with the majority of cases occurring in low- and middle-income countries. We examined the patterns, correlates and care-seeking behaviour of adults suffering from major depressive episode (MDE) in China.

**Method:**

A nationwide study recruited 512 891 adults aged 30–79 years from 10 provinces across China during 2004–2008. The 12-month prevalence of MDE was assessed by the Modified Composite International Diagnostic Interview-short form. Logistic regression yielded adjusted odds ratios (ORs) of MDE associated with socio-economic, lifestyle and health-related factors and major stressful life events.

**Results:**

Overall, 0.7% of participants had MDE and a further 2.4% had major depressive symptoms. Stressful life events were strongly associated with MDE [adjusted OR 14.7, 95% confidence interval (CI) 13.7–15.7], with a dose–response relationship with the number of such events experienced. Family conflict had the highest OR for MDE (18.9, 95% CI 16.8–21.2) among the 10 stressful life events. The risk of MDE was also positively associated with rural residency (OR 1.5, 95% CI 1.4–1.7), low income (OR 2.3, 95% CI 2.1–2.4), living alone (OR 2.6, 95% CI 2.3–3.0), smoking (OR 1.4, 95% CI 1.3–1.6) and certain other mental disorders (e.g. anxiety, phobia). Similar, albeit weaker, associations were observed with depressive symptoms. Among those with MDE, about 15% sought medical help or took psychiatric medication, 15% reported having suicidal ideation and 6% reported attempting suicide.

**Conclusions:**

Among Chinese adults, the patterns and correlates of MDE were generally consistent with those observed in the West. The low rates of seeking professional help and treatment highlight the great gap in mental health services in China.

## Introduction

Globally over 350 million people suffer from major depression (World Health Organization, 2016), which is one of the top 10 causes of years lived with disability in all countries in 2013 (Global Burden of Disease Study 2013 Collaborators, 2015). In China, the recent Global Burden of Disease Study estimated that depression was one of four leading causes of disability-adjusted life-years (DALYs) (Hoevenaar-Blom *et al*. 2014). Despite this, previous cross-country studies have reported that the prevalence of depression was much lower in Chinese than in Western populations (Parker *et al.* 2001), with lifetime rates of major depressive episode (MDE) being only about 1–2% in Chinese (and other East Asians) compared with 10–20% in Western and Middle East populations (Weissman *et al.* 1996; Andrade *et al.* 2003). Several prevalence surveys in mainland China confirmed these estimates, and a recent meta-analysis of 17 studies conducted during 2001–2010 in China reported a mean 12-month MDE prevalence of 2.3% (Gu *et al.* 2013).

Previous studies have also shown consistently that women and individuals with lower socio-economic status had a significantly higher prevalence of depression. However, such factors cannot fully explain the 10-fold variation in prevalence rates of depression across different countries, and other factors such as differences in underlying risk factors, cultural attitudes or health care delivery may also play important roles in the detection and management of MDE in the general population (Weissman *et al.* 1996; Andrade *et al.* 2003). In China, most previous epidemiological studies focused mainly on estimating the prevalence rates, with limited information on correlates or determinants of MDE. Further studies of the patterns, correlates and determinants of MDE in China are needed to provide a better understanding of the disease aetiology and guide the development of population-specific mental health services and prevention programmes.

We analysed relevant data from the China Kadoorie Biobank (CKB) study of 0.5 million adults from 10 diverse urban and rural regions (Chen *et al.* 2005, 2011). The chief objectives of the present study were to: (i) examine the patterns of MDE in men and women across urban and rural regions; (ii) assess comprehensively the associations of MDE with socio-economic, lifestyle and health-related factors as well as with stressful life events and other mental and physical disorders; and (iii) investigate patterns of symptom profiles, care-seeking behaviour and use of psychiatric treatments among those with MDE.

## Method

### Study participants

Details of the CKB design, survey methods and baseline characteristics have been previously reported (Chen *et al.* 2005, 2011). In brief, the CKB covered 1737 communities (rural villages or urban street communities) in 10 regional areas (five urban, five rural) of China, chosen from China's nationally representative Disease Surveillance Points (DSPs). The selection of 10 study sites from DSPs was made carefully, aiming to maximize geographic diversity (including northern and southern regions with very different climate), social diversity (including affluent coastal cities and impoverished inland rural areas) and prevalence of disease patterns (including high rates of stroke in Harbin, oesophageal cancer in Henan or chronic obstructive pulmonary disease in Sichuan), while taking into account population stability, quality of death and disease registries, local commitment and capacity. All 1 801 200 registered permanent residents aged 35–74 years in the study areas were identified through government residential records and invited by door-to-door delivered letters and study information leaflets to attend local study assessment clinics. Overall, 512 891 (including 12 668 just outside this age range) participated from June 2004 to July 2008. As a substantial minority of registered residents would actually be disabled or have been living elsewhere, it was estimated that about a third of the non-disabled invitees actually living in the study areas participated. All participants provided written informed consent. Ethics approval was obtained from the relevant local, national and international authorities.

### Data collection

At the assessment clinics, trained health workers conducted a structured interview using a laptop-based questionnaire (with built-in logic and consistency checks) that covered general demographic (e.g. marital status, household size) and socio-economic status (e.g. income, education, occupation), dietary and other lifestyle habits (e.g. smoking, alcohol drinking, physical activity), sleeping patterns, major stressful life events experienced over the past 2 years, mental status (see below), and prior personal (including history of doctor-diagnosed psychiatric disorders and neurasthenia) and family medical history (including psychiatric disorders of parents and sibling and children). A range of physical measurements (e.g. blood pressure, heart rate, height, weight, waist and hip circumference and lung function) were recorded, and a blood sample was collected for long-term storage (Chen *et al.* 2011). For each participant, the whole survey process lasted about 60–90 min, including 30–45 min for the questionnaire interview.

In each of the 10 study regions, the survey was undertaken by a dedicated team consisting of 15–16 full-time health workers with relevant fieldwork experience. Training on study procedures and methods lasted about 10 days, with formal assessments at various stages. Moreover, the first week of the survey was supervised on site by staff from both the international and national coordinating centres. To ensure data quality and consistency, routine statistical monitoring was performed regularly, both by centre and by individual staff, of the survey data that was transmitted daily to the study coordinating centres.

### Assessment of MDE

MDE was assessed using the Chinese version of the World Health Organization 12-month Composite International Diagnostic Interview short form (CIDI-SF) that included additional questions on suicidal and care-seeking behaviour (Kessler *et al.* 1998; Lee *et al.* 2009).

After approximately 30 min of the interview about their lifestyle and medical history, participants were asked the following four questions: ‘During the past 12 months, have you had the following situations continuously for 2 or more weeks? (i) Feeling much more sad, or depressed than usual; (ii) loss of interest in most things like hobbies or activities that usually give you pleasure; (iii) felt so hopeless that you had no appetite to eat even your favourite food; (iv) feeling worthless and useless, everything went wrong was your fault and life was very difficult that there was no way out’. Participants who answered ‘yes’ to any of the four questions were further assessed using the CIDI-SF (online Supplementary Fig. S1) (Kessler *et al.* 1998).

Those who answered ‘no’ to all four screening questions were classified as ‘screen-negative’ (online Supplementary Fig. S1). Participants who were identified as screen-positive but did not meet CIDI-SF diagnostic criteria were classified as ‘depressive symptoms’ (online Supplementary Fig. S1). At the end of the CIDI, participants were further asked: (*a*) ‘Did you have a plan to harm yourself on purpose during those 2 weeks?’; (*b*) ‘Did you take any action to harm yourself on purpose during those 2 weeks?’ A positive ‘yes’ response to question (*a*) and (*b*) was considered as having suicidal ideation and attempt, respectively.

After the CIDI-SF participants were also asked: (1) ‘Did you tell a doctor about these problems?’; (2) ‘Did you tell any other professional (such as a psychologist, social worker, counsellor, nurse, clergy, or other helping professional working in non-hospital environment)?’; (3) ‘Did you tell your family members or close friends or relatives?’; (4) ‘Did you take medication or use drugs or alcohol more than once for these problems?’; (5) ‘Did you take any treatments for your condition? [including: (*a*) psychiatric treatments; (*b*) herbal medicine; or (*c*) vitamin or other nutritional supplements]’.

General anxiety disorder (GAD) was assessed by the CIDI-SF (B) with the screening question ‘Did you have a period lasting 1 month or longer when most of the time you felt worried, tense, or anxious and it interfered with your life?’. Participants who answered ‘yes’ to the screening question were assessed further by the CIDI-SF (B) and those who met the diagnostic criteria were classified as GAD. All participants were asked to indicate ‘yes’ or ‘no’ to each of the following 10 adverse events during the previous 2 years: (i) marital separation/divorce; (ii) major injury or traffic accident; (iii) loss of job/retirement; (iv) death/major illness of spouse; (v) business failure or bankruptcy; (vi) death/major illness of other close family member; (vii) violence; (viii) major natural disaster (e.g. flood or drought); (ix) major conflict within family; (x) loss of income/living in debt. In addition, information on sleep disorders, panic attacks and phobia (including claustrophobia and agoraphobia) was collected using standard questions at the baseline interview (http://www.ckbiobank.org/site/binaries/content/assets/resources/pdf/qs_baseline-final-from10june2004.pdf).

### Statistical analysis

The mean values and proportions with selected baseline characteristics were calculated by the presence of MDE or depressive symptoms, standardized by 5-year age group, sex and study area. Logistic regression models were used to calculate multivariable-adjusted odds ratios (ORs) of MDE and depressive symptoms for selected baseline variables, including socio-economic status, lifestyle factors, prior history of psychiatric disorders and 10 major stressful life events in the past 2 years. Where appropriate, the analyses were stratified by 5-year age group, sex and study area (10 groups), and all ORs in all tables and figures were adjusted simultaneously for education (four groups: technical school/college/university; middle/high school; primary school; no formal education), household income (four groups: 35 000+; 20 000–34 999; 10 000–19 999; <10 000 yuan per year), occupation (five groups: not in employment; unemployed; office worker; factory worker; agricultural worker), smoking (four groups: never; occasional; ex-regular; current), alcohol (three groups: never; ex-drinker; current), body mass index (BMI) (four groups: <22; 22–24.9; 25.0–26.9; ⩾27.0 kg/m^2^), physical activity [three groups: <15.9; 16.0–31.9; ⩾32 metabolic equivalents (MET)-h/day], prior history of chronic disease (yes/no) and prior history of mental disorders (yes/no).

For any variables involving three or more groups, floating absolute risks were used to provide the variance of the log OR across all categories including the reference group (i.e. those without MDE and depressive symptoms) with a confidence interval (CI) that reflects the amount of data only in that one category (Plummer, 2004). Therefore, even the OR of 1 for the reference group is associated with the variance of the log OR, and hence with a 95% CI. This enables appropriate comparison between any two categories rather than the comparison of a single group with the reference group. All analyses used SAS version 9.3 (SAS Institute, USA).

## Results

**Fig. 1.**
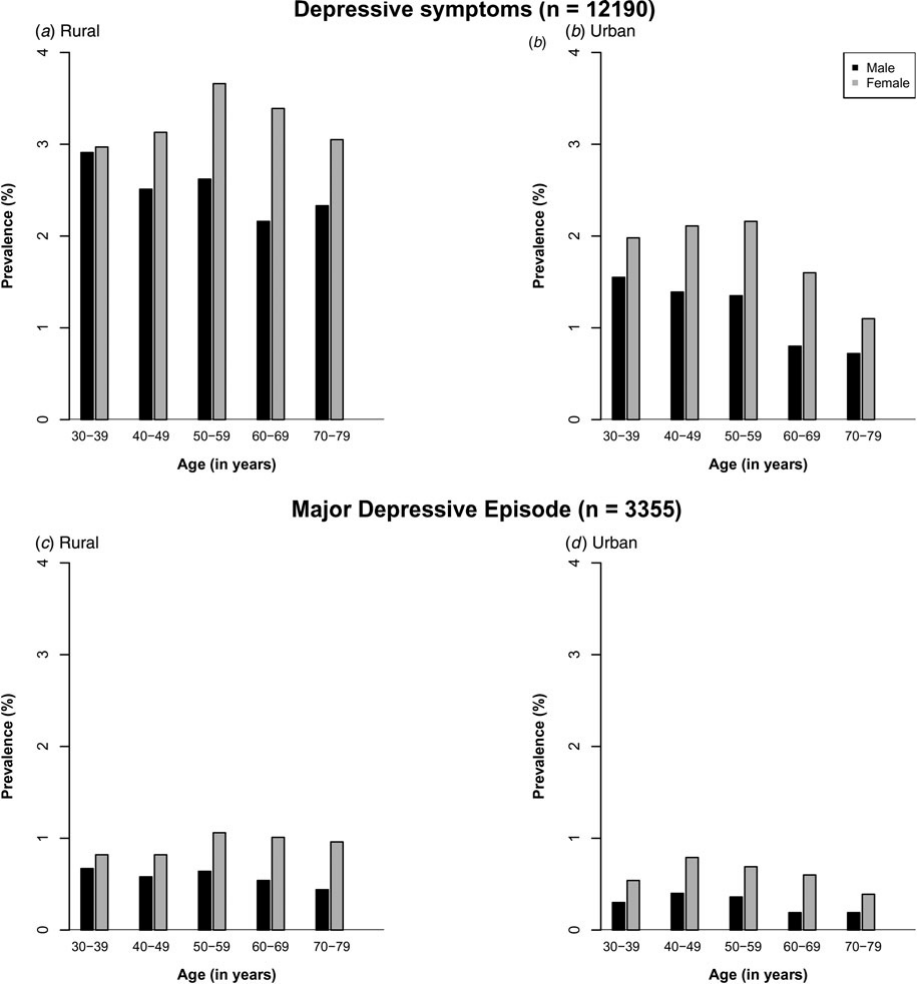
Prevalence of depressive symptoms and major depressive episodes by region, age and sex.

Overall, 0.67% (*n* = 3355) of participants had a MDE and a further 2.39% (*n* = 12 190) had depressive symptoms in the last 12 months. For both, the 12-month rate was higher in women than in men and in individuals living in rural *v*. urban areas (Table 1). Among women, the rates of MDE and depressive symptoms increased with age until about 50 years and fell thereafter; among men they were higher at younger than older age in both rural and urban areas (Fig. 1). The baseline characteristics of individuals with MDE and with depressive symptoms were very similar (Table 1). Compared with screen-negative individuals, those with MDE or depressive symptoms were more likely to have poor education, lower household income, and be unmarried/divorced/widowed or living alone. Moreover, they were also more likely to be current smokers, physically inactive, and to have a family history of mental disorder, self-reported prior chronic diseases and a lower level of self-rated health status and life satisfaction. They also tended to have lower levels of systolic blood pressure, BMI and waist:hip ratio. These associations persisted after adjustments for other relevant covariates (online Supplementary Figs S2 and S3).

**Table 1.** Selected characteristics for all participants^a^

Characteristic	Screened negative (*n* = 497 346)	Depressive symptoms (*n* = 12 190)	Major depressive episode (*n* = 3355)
Mean age, years (s.d.)	51.55 (10.57)	50.98 (10.59)	51.21 (10.58)
Female, %	58.74	66.62	71.37
Rural, %	55.50	69.45	65.57
Socio-economic factors, %			
Less than 6 years education	50.70	53.04	53.52
Annual household income < 10 000 RMB	27.78	42.15	46.36
Currently live alone	2.73	5.34	9.02
Unemployed	2.56	5.12	7.31
Lifestyle factors, %			
Current regular smoker	32.30	34.88	37.85
Problem drinking among current regular drinkers	14.89	13.68	12.72
Current regular drinker	22.38	35.12	29.43
Physical activity < 10 MET-h/day	23.70	27.49	30.09
Physical measurements: mean (s.d.)			
SBP, mmHg	131.84 (20.40)	130.85 (20.43)	130.05 (20.42)
BMI, kg/m^2^	23.67 (3.26)	23.32 (3.26)	23.25 (3.26)
Waist:hip ratio, %	88.19 (6.62)	87.64 (6.63)	87.64 (6.63)
Waist, cm	80.31 (9.28)	78.98 (9.29)	79.15 (9.29)
Prior disease, self-rated health and life satisfaction, %			
Prior chronic disease	23.48	33.67	35.65
Poor health	9.77	27.18	35.75
Unsatisfied with life	3.57	20.74	31.26
Family history of mental disorder, %			
Parent	1.13	2.19	1.91
Sibling	0.51	1.07	1.21
Child	0.21	1.15	1.27
Any member of the family	1.80	4.16	4.12

s.d., Standard deviation; RMB, renminbi; MET, metabolic equivalents; SBP, systolic blood pressure; BMI, body mass index.

a All values are adjusted for age, sex and region where appropriate; all *p* values for heterogeneity <0.0001.

**Fig. 2.**
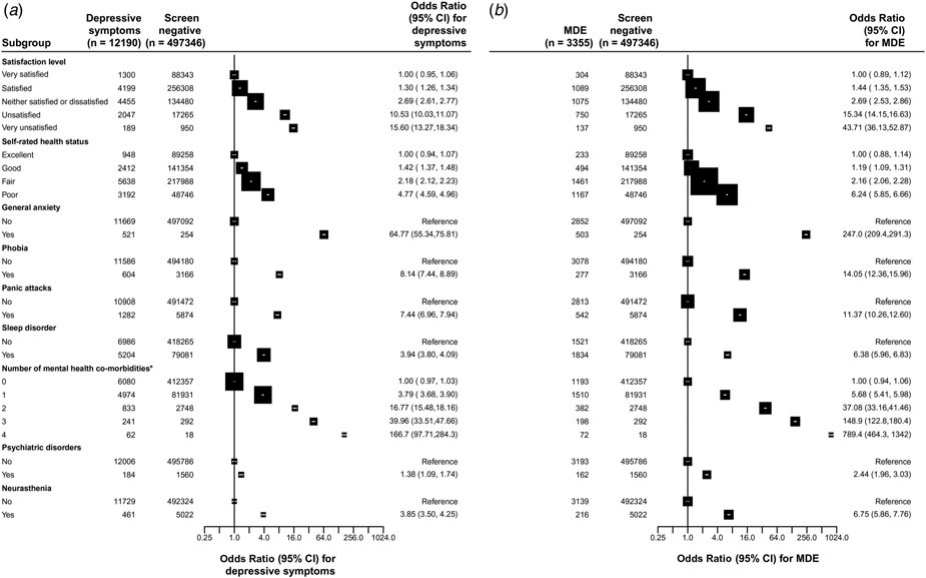
Adjusted odds ratios by health-related symptoms and status for (*a*) depressive symptoms and (*b*) major depressive episode (MDE). Each closed square represents an odds ratio and the size of the squares is inversely proportional to the variance of the log odds ratio in that group, after taking account of the variance of the log risk in the reference group. The horizontal lines represent the 95% confidence interval (CI). All odds ratios were adjusted for age, sex, region, income, education, occupation, body mass index, physical activity, and prior chronic disease including mental disorders where appropriate. *Participants with a combination of anxiety, phobia, panic attacks and sleep disorder.

Fig. 2 shows the adjusted ORs for depressive symptoms (*a*) and MDE (*b*) by self-rated life satisfaction and health status, along with a range of co-morbid chronic mental disorders, family history of psychiatric disorders or neurasthenia. Compared with those who were very satisfied with life, the adjusted ORs of MDE increased with the reduced degree of self-rated life satisfaction, with ORs of 1.44 (95% CI 1.35–1.53), 2.69 (95% CI 2.53–2.86), 15.34 (95% CI 14.15–16.63) and 43.71 (95% CI 36.13–52.87), respectively, for those who reported being satisfied, neither satisfied nor dissatisfied, unsatisfied, very unsatisfied with life compared with those who were very satisfied. Similarly, compared with those with excellent self-rated health status, the adjusted ORs were 1.19 (95% CI 1.09–1.31), 2.16 (95% CI 2.06–2.28) and 6.24 (95% CI 5.85–6.66), for those with good, fair and poor health status, respectively. The adjusted ORs of MDE were also strongly associated with several self-reported co-morbid psychiatric disorder or conditions (Fig. 2*b*), including GAD (OR 247.0, 95% CI 209.4–291.3), phobia (OR 14.05, 95% CI 12.36–15.96), panic attacks (OR 11.37, 95% CI 10.26–12.60) and sleep disorders (OR 6.38, 95% CI 5.96–6.83). A prior history of a psychiatric disorder was associated with an OR of 2.44 (95% CI 1.96–3.03) and neurasthenia was associated with an OR of 6.75 (95% CI 5.86–7.76) for MDE. The ORs also increased progressively with the number of these co-morbid psychiatric conditions, with adjusted ORs of 5.68 (95% CI 5.41–5.98), 37.08 (95% CI 33.16–41.46), 148.9 (95% CI 122.8–180.4) and 789.4 (95% CI 464.3–1342.0) for those with one, two, three and four such mental conditions, respectively. Individuals who solely had depressive symptoms had similar, albeit more modest, associations with these risk factors. Likewise, the associations with depressive symptoms were similar to those for MDE, albeit the ORs were less extreme (Fig. 2*a*).

**Fig. 3.**
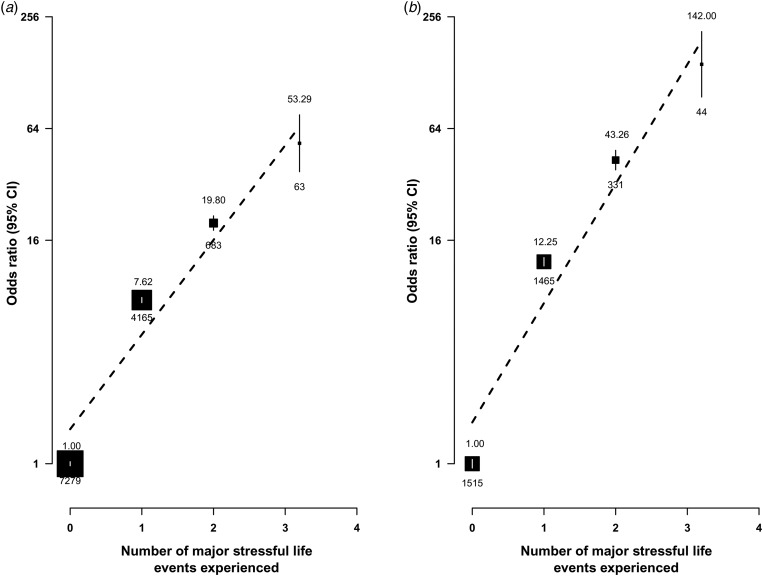
Adjusted odds ratio (OR) by number of stressful life events experienced for (*a*) depressive symptoms and (*b*) major depressive episodes. The number above the black box indicates OR and the number beneath it indicates the number of participants. The symbols and conventions are the same as those used in Fig. 2. The vertical bars represent the 95% confidence interval (CI).

Table 2 shows the adjusted ORs of depressive symptoms and MDE, by types of major stressful life events occurring during the last 2 years. The adjusted OR of depressive symptoms for individuals who had experienced any stressful life events (OR 8.47, 95% CI 8.15–8.81) was less extreme than that for MDE (OR 14.68, 95% CI 13.68–15.75). Of the 10 major stressful life events surveyed, family-related events (e.g. divorce or separation, family conflict, or death of spouse), loss of income or debt and violence were each associated with an OR of 10 or greater for MDE and a somewhat smaller OR for depressive symptoms. With the exception of job loss or retirement, other stressful life events were also associated with ORs of 5 to 10 for MDE. Moreover, the ORs increased linearly and progressively with the number of stressful life events experienced during the previous 2 years, with those experiencing three or more events having an OR of 53.3 (95% CI 32.4–75.8) for depressive symptoms and 142.0 (95% CI 94.6–213.1) for MDE (*p* < 0.0001 for trend for both; Fig. 3).

**Table 2.** Adjusted odds ratios for depressive symptoms and major depressive episode by types of major stressful life events

Type of event	Screened negative (*n* = 497 346)		Depressive symptoms (*n* = 12 190)		Major depressive episode (*n* = 3355)	
	No. without life events	No. with life event	No. with life event	Odds ratio (95% CI)^a^	No. with life event	Odds ratio (95% CI)^a^
Family-related events						
Divorce or separation	496 291	1055	205	8.67 (7.43–10.12)	85	12.15 (9.66–15.30)
Family conflict	494 195	3151	987	13.28 (12.31–14.33)	389	18.88 (16.85–21.17)
Death of spouse	493 799	3547	727	9.37 (8.61–10.20)	392	18.70 (16.65–21.00)
Death or major illness in close family member	474 939	22 407	2393	5.06 (4.83–5.31)	888	7.32 (6.77–7.93)
Finance-related events						
Loss of income or debt	495 303	2043	475	8.17 (7.36–9.06)	198	11.53 (9.87–13.46)
Job loss or retirement	495 342	2004	207	5.06 (4.36–5.87)	55	3.81 (2.89–5.02)
Bankruptcy	496 408	938	197	7.20 (6.15–8.44)	66	8.70 (6.73–11.25)
Other events						
Violence	496 802	544	117	7.95 (6.49–9.75)	54	12.68 (9.51–16.90)
Major injury or traffic accident	494 534	2812	368	5.10 (4.56–5.70)	126	6.29 (5.23–7.56)
Natural disaster	496 944	402	61	5.46 (4.15–7.18)	15	4.98 (2.96–8.37)
Any major stressful life event	460 645	36 701	4911	8.47 (8.15–8.81)	1840	14.68 (13.68–15.75)

CI, Confidence interval.

a All values are adjusted for age at baseline, sex, region, education, income, occupation and prior history of chronic disease.

Table 3 shows the symptom profile and help-seeking behaviours among the 3355 participants with MDE. Overall, about 80% or more participants reported having loss of interest, insomnia, tiredness and difficulty in concentration, and that the MDE had significantly interfered with their life. Moreover, 15% reported having suicidal ideation and 6% a suicidal attempt, both being more common in women than in men. Of those with MDE, 61% (women 64%, men 52%) sought any help, mainly from families or friends (52%) rather than from the medical profession (15%). Among those reporting suicidal ideation and suicidal attempt, medical help was sought by 21% and 11%, respectively (data not shown). Less than 10% were taking specific psychiatric medications for treatment of MDE (35% and 15% among those reporting suicidal ideation and suicidal attempt, respectively, data not shown). Compared with women, men with a MDE were twice as likely to use alcohol and drugs (18% *v*. 8%).

**Table 3.** Distribution of self-reported symptoms and disease management for major depressive episode by sex^a^

	Males (*n* = 975): *n* (%)	Females (*n* = 2380): *n* (%)	Total (*n* = 3355): *n* (%)
Symptoms			
Loss of interest	857 (87.90)	2141 (89.96)	2998 (89.36)
Tiredness	768 (78.77)	1941 (81.55)	2709 (80.75)
Weight gain	52 (5.33)	172 (7.23)	224 (6.68)
Weight loss	395 (40.51)	1133 (47.61)	1528 (45.54)
Insomnia	794 (81.44)	2023 (85.00)	2817 (83.96)
Feelings of worthlessness	437 (44.82)	1115 (46.85)	1552 (46.26)
Indecision and difficulty concentrating	764 (78.36)	1921 (80.71)	2685 (80.03)
Suicidal ideation	125 (12.82)	381 (16.01)	506 (15.08)
Suicide attempt	50 (5.13)	156 (6.55)	206 (6.14)
Significant inference with life	784 (80.41)	1961 (82.39)	2745 (81.82)
Sought help from			
Any source	505 (51.79)	1527 (64.16)	2032 (60.57)
Doctor	129 (13.23)	370 (15.55)	499 (14.87)
Other professional	260 (26.67)	828 (34.79)	1088 (32.43)
Family or friend	421 (43.18)	1324 (55.63)	1745 (52.01)
Using or receiving medication			
Drugs or alcohol	172 (17.64)	197 (8.28)	369 (11.00)
Psychiatric treatment	86 (8.82)	211 (8.87)	297 (8.85)
Herbal medicine	53 (5.44)	180 (7.56)	233 (6.94)
Vitamins or other health products	83 (8.51)	271 (11.39)	354 (10.55)

a All percentages are adjusted for age and region.

## Discussion

In this large community-based study in China, about 1% of participants had MDE in the last 12 months and a further 2% experienced major depressive symptoms. Despite the relatively low prevalence compared with 2.3% from a meta-analysis of 17 studies in China (Gu *et al.* 2013), the characteristics and patterns of associations of MDE with a range of socio-economic, lifestyle and health-related factors were broadly similar to those previously reported in Western populations. Moreover, a high proportion (15%) of individuals with MDE reported having suicidal ideation and 6% reported having an attempted suicide, but less than 10% received any relevant psychiatric treatments. Importantly, recent major stressful life events, including family-related conflict, financial difficulties, violence, injury and natural disasters, were significantly associated with higher ORs of both MDE and depressive symptoms.

### Major stressful life events

Consistent with previous studies in the West (Kessler, 1997; Kendler *et al.* 1998), we reported a strong positive association of recent stressful life events in the last 2 years with MDE and in those with depressive symptoms. Furthermore, the risks of both MDE and depressive symptoms increased linearly with the number of stressful life events, with those who had experienced three or more such events having more than 50-fold and 140-fold higher prevalence of depressive symptoms and MDE, respectively. A recent study (CONVERGE) in China found that stress caused changes in both mitochondrial DNA and telomere length, both being significantly associated with MDE (Cai *et al*. 2015; CONVERGE Consortium, 2015). In the present study, over one-fifth of stressful life events in the most recent 2 years were due to conflicts within families (Table 2), which, compared with loss of income or being in debt (OR 11.53) or major injury or traffic accident (OR 6.29), had much higher risk of MDE (OR 18.88). Previously only a few small studies had reported on the association of family conflict with depressive symptoms, including a cross-sectional study of 385 Chinese Americans aged 55 years and over, a longitudinal study of 1527 antenatal Chinese women (Lau, 2011; Sun *et al.* 2016) and a Mexican study of 262 adolescents (Gil-Rivas *et al.* 2003). The present study has demonstrated strong association of family conflict with MDE and with depressive symptoms in both men and women. The nature of family conflicts and their association with MDE needs to be investigated in detail in future studies. Previous studies in China have also shown acute interpersonal conflict to be associated with higher suicide rates (Phillips *et al.* 2002; Yang *et al.* 2005). The current social support system in China is strongly dependent on family members for help. The finding of the present study suggests that providing alternative social support in the community and managing acute family and interpersonal conflicts are important in the prevention of MDE as well as suicide (Phillips *et al.* 2002; Yang *et al.* 2005).

### Socio-economic and lifestyle factors and urban–rural differences

The present study, including over 500 000 individuals from five urban and five rural regions of China, reported higher risks of both MDE and depressive symptoms associated with low socio-economic status (low education, low household income, unemployment), regular smoking and low BMI. This is largely consistent with previous studies in the West (Glassman *et al.* 1990; Kendler *et al.* 1993, 1996). Compared with their urban counterparts, residents in rural areas had 54% higher risks of MDE, somewhat greater than in a previous meta-analysis of 17 studies in China (Gu *et al.* 2013). Despite the similar finding in a small study in Singapore (Lim & Kua, 2011), the present study is perhaps the first large study to reliably demonstrate that living alone is also an important correlate of MDE in China. As the population in China is ageing and the proportion living alone is increasing, particularly in response to the one-child policy, the findings in the present study have important implications for mental health policy in China. Future studies in the CKB will further investigate whether the observed rural–urban difference can be attributed to the differences in socio-economic status, frequency of stressful life events, living alone, mental health status or other region-specific risk factors.

### Mental and physical wellbeing

Like previous studies of Western populations (Kessler *et al.* 2003), we have also demonstrated strong associations of MDE with other psychiatric disorders (including GAD, phobias, panic symptoms and sleep disorders). The risks of both MDE and depressive symptoms increased linearly with the number of co-morbid psychiatric disorders. Interestingly, self-reported doctor-diagnosed neurasthenia was associated with a 60% higher risk of MDE, lending support to the hypothesis that many Chinese tend to manifest depression through physical symptoms (Parker *et al.* 2001).

The association of life satisfaction and depression has been examined in a few small studies (Lacruz *et al.* 2016; Yazdanshenas Ghazwin *et al.* 2016). The present study also demonstrated that both self-rated life satisfaction status and self-rated health status were linearly associated with the risk of MDE and depressive symptoms in the Chinese population (Fig. 2). Moreover, individuals who rated themselves as being unsatisfied with life had a 7-fold higher risk of MDE than those who rated themselves in poor health status, suggesting that mental health may be more important than physical wellbeing for the prediction and prevention of MDE. Given the high co-morbidity of MDE with other mental disorders including anxiety, MDE should be treated within the package of mental disorders rather than considered alone. Importantly, the present study suggests that a simple question of life satisfaction is effective for identifying individuals at high risk of MDE and that such questions should be included in future large epidemiological studies on health and disease in the general population.

### Depressive symptoms *v*. depressive disorder

Previous studies, have evaluated the prevalence of depressive symptoms to assess mood problems without subsequent examination to estimate the prevalence of MDE. In the present study the prevalence of depressive symptoms was about three times as high as that of MDE. Despite this, depressive symptoms shared almost identical, albeit more modest, risk profiles to those for MDE. Hence, mood disorders such as MDE should be viewed as a continuum rather than a dichotomous condition and ‘depressive symptoms’ may be considered as a pre-MDE status. Future studies should examine whether early intervention in people with depressive symptoms could prevent these from developing MDE and whether the long-term outcomes of participants with depressive symptoms differ significantly from those participants with MDE. Compared with diagnostic instruments used for MDE, which require specialized training and a lengthy interview, the inclusion of four simple screening questions in the questionnaire was an effective alternative approach to identify people at high risk of MDE in large-scale studies.

### Care seeking and mental health services

Previous large epidemiological study on mental disorder during 2001–2005 reported that about 90% of individuals with mental disorders in China had never sought any type of professional help (Phillips *et al.* 2009). In the present study, although 60% of study participants with MDE sought help, only 15% sought help from a medical professional, consistent with previous estimates. Of those with MDE, only 9% reported current use of psychiatric medications, compared with over a half of the MDE cases in the USA (Kessler *et al.* 2003). Apart from stigma-induced unwillingness to seek professional help, lack of effective mental health services including provision of medication in rural areas, lack of training of community-based health professionals and reluctance of many health professionals to provide psychological services may explain the low use of mental health services (Phillips, 2013). The introduction of China's first Mental Health Law in 2012 (Eleventh National People's Congress Standing Committee, 2012), which sought to expand access to mental health services by shifting the focus from specialized psychiatric hospitals to general hospitals and community health clinics in both rural and urban areas, is a positive national policy response to reduce the substantial burden of mental illness in China. However, more qualitative studies are needed to examine factors that influence care-seeking for MDE such as differences in infrastructure and available resources between urban and rural regions, and cohort-specific studies on interventions that increase care-seeking are urgently needed.

### Strengths and limitations

The chief strengths of the present study were the large sample size and diverse regions involved, the use of internationally validated instruments for assessing depressive episodes and other psychiatric symptoms, and directly recorded physical measurements. Moreover, the information collected covered socio-economic, health-related behaviour characteristics, and physical and mental health status, which enabled a comprehensive assessment in a single study of their associations with MDE. The study also has a number of limitations. First, the 12-month prevalence rates (0.7%) observed in the present study were about one-third of those (2.3%) reported in a recent meta-analysis of 17 studies in China (Gu *et al*. 2013). Contrary to these previous studies that mainly covered the typical onset age of MDE (i.e. 15–30 years), the CKB covered a much older population (i.e. aged 35–74) in order to assess the main determinants of common chronic diseases (e.g. stroke, cancer, heart disease, diabetes or chronic respiratory diseases). Moreover, study participation was on a voluntary basis and individuals who were suffering from MDE or had a severe MDE may have been less likely to participate in the CKB, particularly when the response rate was low for estimating reliably the prevalence rates. Hence, the present study has almost certainly underestimated the 12-month prevalence rate of MDE. Despite the low prevalence, the symptom profile, patterns and correlates of MDE observed in the present study are remarkably consistent with previously published estimates in China (Gu *et al.* 2013) and in Western populations (Kessler *et al.* 1994; Sullivan *et al.* 1998; Kendler *et al.* 2015). The large sample size, inclusion of large numbers from diverse communities across China and consistent findings across different population subgroups should permit generalizability of the present study findings to the population at large, at least those with similar age range. Second, MDE could occur after physical illness or other mental disorders such as anxiety. The CIDI-SF instrument used in the present study cannot distinguish primary and secondary MDE. Despite the fact that the baseline survey was carried out in an apparently healthy population and individuals with acute medical or psychiatric conditions were less likely to attend the face-to-face interview, we cannot exclude the possibility that some of MDE may be related to chronic diseases occurring prior to the survey. Nonetheless, the lack of any material changes in the results in sensitivity analyses among individuals without prior physical or psychiatric disorders suggested that MDE identified in the present study is more likely to be primary rather than due to other diseases. Third, as all the analyses were based on cross-sectional rather than on prospective data, we cannot fully exclude the effects of reverse causality. For example, some of the stressful life events could have followed rather than preceded the depressive episodes.

In conclusion, the present study findings have several important implications for prevention and mental health policy. First, in China and other developing countries with limited health resources, the efforts should be prioritized toward high-risk populations (e.g. women, rural residents and individuals with lower levels of socio-economic status). Second, recent stressful life events, in particular conflicts within the family, are an important determinant of MDE. Hence, it is important for mental health services to provide alternative social support and effective management of interpersonal conflicts in the community. Third, the present study demonstrated that psychological wellbeing is more important than physical wellbeing for the prevention of MDE. Moreover, MDE should be treated together with co-morbid mental disorders. Fourth, screening of depressive symptoms could be a very effective strategy for the early detection and prevention of MDE. Importantly, the low use of help-seeking behaviour and low use of treatments highlight the substantial unmet needs and emphasize the importance of studying effective ways to deliver mental health services as well as identifying barriers to access to professional care among individuals with either MDE or with depressive symptoms in China.

## Supplementary material

The supplementary material for this article can be found at https://doi.org/10.1017/S0033291716002889

## Appendix. China Kadoorie Biobank Collaborative Group

**International Steering Committee:** Junshi Chen, Zhengming Chen (principal investigator), Rory Collins, Liming Li (principal investigator), Richard Peto.

**International Co-ordinating Centre, Oxford:** Daniel Avery, Derrick Bennett, Yumei Chang, Yiping Chen, Zhengming Chen, Robert Clarke, Huaidong Du, Simon Gilbert, Alex Hacker, Michael Holmes, Andri Iona, Christiana Kartsonaki; Rene Kerosi, Ling Kong, Om Kurmi, Garry Lancaster, Sarah Lewington, John McDonnell, Iona Millwood, Qunhua Nie, Jayakrishnan Radhakrishnan, Paul Ryder, Sam Sansome, Dan Schmidt, Paul Sherliker, Rajani Sohoni, Iain Turnbull, Robin Walters, Jenny Wang, Lin Wang, Ling Yang, Xiaoming Yang.

**National Co-ordinating Centre, Beijing:** Zheng Bian, Ge Chen, Yu Guo, Bingyang Han, Can Hou, Jun Lv, Pei Pei, Shuzhen Qu, Yunlong Tan, Canqing Yu, Huiyan Zhou.

**10 Regional co-ordinating centres**

**Qingdao** Qingdao CDC: Zengchang Pang, Ruqin Gao, Shaojie Wang, Yongmei Liu, Ranran Du, Yajing Zang, Liang Cheng, Xiaocao Tian, Hua Zhang. Licang CDC: Silu Lv, Junzheng Wang, Wei Hou.

**Heilongjiang** Provincial CDC: Jiyuan Yin, Ge Jiang, Shumei Liu, Zhigang Pang, Xue Zhou. Nangang CDC: Liqiu Yang, Hui He, Bo Yu, Yanjie Li, Huaiyi Mu, Qinai Xu, Meiling Dou, Jiaojiao Ren.

**Hainan** Provincial CDC: Jianwei Du, Shanqing Wang, Ximin Hu, Hongmei Wang, Jinyan Chen, Yan Fu, Zhenwang Fu, Xiaohuan Wang, Hua Dong. Meilan CDC: Min Weng, Xiangyang Zheng, Yijun Li, Huimei Li, Chenglong Li.

**Jiangsu** Provincial CDC: Ming Wu, Jinyi Zhou, Ran Tao, Jie Yang. Suzhou CDC: Jie Shen, Yihe Hu, Yan Lu, Yan Gao, Liangcai Ma, Renxian Zhou, Aiyu Tang, Shuo Zhang, Jianrong Jin.

**Guangxi** Provincial CDC: Zhenzhu Tang, Naying Chen, Ying Huang. Liuzhou CDC: Mingqiang Li, Jinhuai Meng, Rong Pan, Qilian Jiang, Jingxin Qing, Weiyuan Zhang, Yun Liu, Liuping Wei, Liyuan Zhou, Ningyu Chen, Jun Yang, Hairong Guan.

**Sichuan** Provincial CDC: Xianping Wu, Ningmei Zhang, Xiaofang Chen, Xuefeng Tang. Pengzhou CDC: Guojin Luo, Jianguo Li, Xiaofang Chen, Jian Wang, Jiaqiu Liu, Qiang Sun.

**Gansu** Provincial CDC: Pengfei Ge, Xiaolan Ren, Caixia Dong. Maiji CDC: Hui Zhang, Enke Mao, Xiaoping Wang, Tao Wang.

**Henan** Provincial CDC: Guohua Liu, Baoyu Zhu, Gang Zhou, Shixian Feng, Liang Chang, Lei Fan. Huixian CDC: Yulian Gao, Tianyou He, Li Jiang, Huarong Sun, Pan He, Chen Hu, Qiannan Lv, Xukui Zhang.

**Zhejiang** Provincial CDC: Min Yu, Ruying Hu, Le Fang, Hao Wang. Tongxiang CDC: Yijian Qian, Chunmei Wang, Kaixue Xie, Lingli Chen, Yaxing Pan, Dongxia Pan.

**Hunan** Provincial CDC: Yuelong Huang, Biyun Chen, Donghui Jin, Huilin Liu, Zhongxi Fu, Qiaohua Xu. Liuyang CDC: Xin Xu, Youping Xiong, Weifang Jia, Xianzhi Li, Libo Zhang, Zhe Qiu.
